# Mobile phone ownership among young adults in seven Southern African countries

**DOI:** 10.7189/jogh.15.04123

**Published:** 2025-04-18

**Authors:** Abigail R Greenleaf, Monique Millington, Laura Robles-Torres, Fred Asiimwe, Huguette Diakabana, Sarah D Francis, Tendayi Mharadze, Jessica Justman

**Affiliations:** 1 Mailman School of Public Health – Columbia University, ICAP at Columbia University, New York, New York, USA; 2 Mailman School of Public Health – Columbia University Heilbrunn Department of Population and Family Health, New York, New York, USA; 3 City University of New York, School of Public Health & Health Policy, New York, New York, USA; 4 Centers for Disease Control and Prevention, Division of Global HIV and TB, Center for Global Health, Maseru, Lesotho; 5 African Foundation for Peace and Security – Johannesburg, South Africa; 6 Mailman School of Public Health – Columbia University, Department of Epidemiology, New York, New York, USA; 7 Centers for Disease Control and Prevention, Division of Global HIV and TB, Center for Global Health, Harare, Zimbabwe

## Abstract

**Background:**

In sub-Saharan Africa, mHealth interventions and phone-based data collection are increasingly popular but little is known about who can be reached by these programmes. We used national probability surveys to examine characteristics of youth (15–24 years) mobile phone owners in seven Southern African countries: Botswana, Eswatini, Lesotho, Malawi, Mozambique, Zambia, and Zimbabwe.

**Methods:**

Population-based HIV Impact Assessment surveys are cross-sectional, nationally representative household-based surveys conducted between November 2019 and February 2022. Data were analysed using multivariable logistic regression.

**Results:**

Eighty-four percent of youth in Eswatini, 83% in Botswana, 76% in Lesotho, 61% in Zimbabwe, 47% in Mozambique, 46% in Zambia and 32% in Malawi were mobile phone owners. In all countries, odds of phone ownership were higher amongst persons ages 20–24 (compared to 15–19) and those with secondary education or higher. In the three countries with ownership less than 50%, women had lower odds of owning a phone than men, and all wealth quintiles had higher odds of ownership than the lowest wealth quintile.

**Conclusions:**

Mobile phone ownership was consistently higher among certain demographic groups. Public health practitioners employing mobile phones for youth health programmes in Sub-Saharan Africa may not reach the general youth population.

In the last decade, sub-Saharan Africa (SSA) experienced rapid growth in mobile phone ownership from 32% of adults in 2012 to currently almost 50% ownership [1]. Over the next five years, 167 million new subscribers will join the market [2]. The majority of these new subscribers will be youth who constitute the demographic dividend in SSA and have grown up with technology. Those born between the mid-1990s and the early 2010s will not only drive mobile phone ownership, but will change the way mobile phones are used. Young people have different mobile phone usage patterns than older generations: they are more likely to have smartphones, access the internet and use social media [3].

Global health practitioners have targeted young people via mobile phones to collect data, stage behaviour change communication campaigns, and monitor health programmes [4]. A recent systematic review established that in SSA, mHealth (use of technology/computing, such as phones, to enable health care and programmes) interventions improved adolescent uptake of sexual and reproductive health services, most consistently for contraceptive use [5]. mHealth programmes are also frequently used along the HIV continuum of care (diagnosing, engaging in care and adherence to effective medication), with high rates of acceptability but mixed effectiveness [6]. Such programmes range from virtual peer mentorship for youth recently diagnosed with HIV in South Africa [7] to a sexual education app in Zambia [8] and text messaging-based HIV prevention programmes for sexually active young adults in Uganda [9].

Although mHealth interventions and phone-based data collection are increasingly popular, barriers such as lack of mobile phone availability and access, technology gaps and insufficient infrastructure limit the effectiveness of mHealth interventions [10]. Research on mobile phone ownership generally shows demographic differences between mobile phone owners and non-owners. Phone owners are often younger, male, more educated, wealthier and urban compared to those who do not own a phone [11–15]. Recent findings assert that among women in SSA, phone ownership is positively associated with indicators of global social development, such as high contraceptive use, and lower gender inequality, and lower maternal and child mortality [16].

For an mHealth intervention to be successful, those targeted for the intervention must own or have access to a phone. However, the handful of studies about youth phone ownership in SSA are insufficient to plan public health programmes. We used data from the Population-based HIV Impact Assessment (PHIA) – a cross-sectional national probability household-based survey to examine characteristics of young (15–24 years) mobile phone owners in seven Southern African Development Community countries [17]: Botswana, Eswatini, Lesotho, Malawi, Mozambique, Zambia, and Zimbabwe.

## METHODS

### Survey

Population-based HIV Impact Assessment surveys were conducted in Botswana (2021), Eswatini (2021), Lesotho (2019–2020), Malawi (2020–21), Mozambique (2021–22) Zambia (2021), and Zimbabwe (2019–2020) between November 2019 and February 2022 to obtain nationally representative measures of HIV incidence and prevalence, and to evaluate the status of HIV treatment and care. All questionnaires were interviewer-administered and depending on country procedures, interviewers obtained either verbal or written informed consent/assent for the interview. Consenting heads of households completed a household questionnaire and listed household members and guests who had slept in the household the previous night. All children and adults aged 15 and above (except Botswana, aged 15–64 years) from selected households were invited to participate in the survey. Interviewers obtained consent from parents or guardians to approach those aged 15–17 years who then assented to participate. All household response rates were above 84%; interview and blood draw response rates ranged between 63% (Malawi) and 84% (Eswatini). Detailed information on study design, sampling and response rates are available elsewhere [18,19].

The surveys were reviewed and approved by the Columbia University (Eswatini, Lesotho, Malawi, Mozambique and Zimbabwe), University of Maryland – Baltimore (Botswana and Zambia), Centers for Diseases Control and Prevention, and local Institutional Review Boards (see Health and Human Services’ Office for Human Research Protections 45 C.F.R. part 46; 21 C.F.R. part 56, https://www.hhs.gov/ohrp/regulations-and-policy/regulations/45-cfr-46/index.html).

### Sample characteristics

This analysis was conducted using the de-identified data of participants aged 15–24 years in all countries except Zambia and Botswana (where we included only those 16–24 years as the parents of those aged 15 were asked these questions rather than the youth themselves) who slept in the household the night before and consented or assented to the individual interview then HIV testing.

### Measurement of key variables

The outcome for this analysis was mobile phone ownership. All participants who consented/assented to HIV testing or who agreed to future contact for research were asked ‘Can we attempt to contact you by phone?’. Participants who responded ‘no’ were classified as ‘non phone owners.’ Participants who responded ‘yes’ were asked to provide a mobile phone number. If a mobile number was provided, participants were asked to identify whether the phone was owned by themselves, a parent/guardian, or someone else. Participants who indicated that they owned the phone were defined as ‘phone owners’ and those who gave the phone number of someone else were defined as having ‘phone access.’ Given that mobile phone ownership is not increasing rapidly in these countries, countries are comparable despite being collected over three years (2019–2022).

Independent variables that were self-reported include age (categorised into two groups: 15–19 (16–19 Botswana and Zambia), 20–24 years), gender (male *vs*. female), marital status (currently not married *vs*. married or living together), and highest school attended (no education, primary, secondary, more than secondary; and as two groups for the regression: less than secondary, secondary or higher). Residence (urban *vs*. rural) was pre-determined by the local statistical agency, and household wealth quintile (five groups) was created using principal components analysis for each country. Staff conducted rapid HIV testing using each country’s national three-test (Zimbabwe) or two test (all other countries) clinical algorithm [19]. Laboratory confirmation of HIV-positive results was done using the Geenius HIV 1/2 supplemental assay (Bio-Rad, USA). For regression analyses, the outcome of interest was phone ownership *vs*. phone access or no phone.

### Statistical analysis

To provide practitioners with results specific to their country context, analyses were country-specific (*i.e*. not pooled) and conducted using STATA, version 18.0 (StataCorp LLC, College Station, Texas, USA). The final PHIA survey weights accounted for survey design, non-response rates, and poststratification. To assess the association between the independent and outcome variables, we used the survey-adjusted Wald test to create descriptive results. Covariates that were significantly associated (*P* ≤ 0.05) with mobile phone ownership in bivariate analyses or had empirical precedence were included in the final multivariable logistic regression except marriage, which was not statistically significant in all countries and due to a small percent of young people being married in a few countries, resulted in large confidence intervals. All counts are unweighted and point estimates are weighted with standard errors derived using Jackknife replicates.

## RESULTS

### Sample characteristics

The final samples for this analysis consisted of participants ages 15–24 years from Botswana (n = 3607; 16–24 years), Eswatini (n = 3259), Lesotho (n = 4211), Malawi (n = 8266), Mozambique (n = 4803), Zambia (n = 5552; 16–24 years), Zimbabwe (n = 5949) (**Table 1**). The mean age was approximately 19 years in all countries. Urban residence ranged from 68% for the Botswana youth to 19% for the Malawi youth. Mozambique had the greatest proportion of youth who were married or living together (34%), and Eswatini had the least (5%). Botswana had the highest proportion of youth with secondary or higher education (95%) while Malawi had the least (33%). In all countries except Mozambique, the 15–24 population was evenly distributed between households in the five wealth quintiles (within five percentage points of 20%). Mozambique had more youth in the highest wealth quintile (28%) and fewer in the lowest (14%).

**Table 1:** Characteristics of nationally representative sample of young people in seven Southern Africa countries, 2020–2022

Variables	Botswana	Eswatini	Lesotho	Malawi	Mozambique	Zambia	Zimbabwe
	**(n = 3607)**	**(n = 3259)**	**(n = 4211)**	**(n = 8266)**	**(n = 4803)**	**(n = 5552)**	(n = 5949)
**Age in years, mean (SD)**	19.919 (2.536)	19.524 (2.753)	19.475 (2.785)	19.205 (2.768)	19.260 (2.695)	19.706 (2.584)	19.324 (2.739)
**Age group**							
15–19*	1608 (45.9%)	1974 (53.6%)	2075 (51.0%)	4070 (54.1%)	2360 (54.4%)	2640 (49.1%)	3207 (54.0%)
20–24	1999 (54.1%)	1660 (46.4%)	2136 (49.0%)	4196 (45.9%)	2443 (45.6%)	2912 (50.9%)	2742 (46.0%)
**Gender**							
Male	1595 (51.6%)	1576 (51.1%)	1744 (50.0%)	3691 (48.6%)	2132 (48.9%)	2132 (48.9%)	2582 (49.7%)
Female	2012 (48.4%)	1683 (48.9%)	2467 (50.0%)	4575 (51.4%)	2671 (51.1%)	2671 (51.1%)	3367 (50.3%)
**Residence**							
Rural	1517 (32.0%)	2682 (72.4%)	2087 (48.0%)	6575 (80.7%)	2518 (55.5%)	3321 (57.2%)	4192 (68.0%)
Urban	2090 (68.0%)	577 (27.6%)	2124 (52.0%)	1691 (19.3%)	2285 (44.5%)	2231 (42.8%)	1757 (32.0%)
**Highest school attended**							
No education	60 (2.0%)	20 (0.6%)	71 (1.8%)	218 (2.6%)	427 (10.1%)	224 (3.7%)	35 (0.6%)
Primary	112 (2.7%)	455 (13.5%)	946 (22.5%)	5258 (64.1%)	1887 (41.5%)	1725 (29.6%)	1507 (23.9%)
Secondary	2860 (75.4%)	2544 (77.6%)	2859 (67.6%)	2577 (31.0%)	2374 (46.6%)	3434 (62.8%)	4165 (71.2%)
More than secondary	575 (19.9%)	240 (8.3%)	335 (8.1%)	209 (2.4%)	111 (1.8%)	167 (3.8%)	237 (4.3%)
**Wealth quintile**†							
Lowest	876 (17.0%)	721 (19.9%)	929 (20.4%)	1415 (17.5%)	589 (13.9%)	1371 (17.9%)	1371 (20.0%)
Second	827 (22.1%)	923 (25.8%)	875 (20.9%)	1569 (18.7%)	738 (16.7%)	1119 (18.0%)	1271 (20.6%)
Middle	620 (20.1%)	692 (20.8%)	864 (20.8%)	1614 (19.5%)	765 (16.5%)	924 (19.1%)	1192 (20.8%)
Fourth	674 (20.9%)	495 (16.9%)	831 (20.4%)	1781 (21.9%)	1156 (24.7%)	1046 (21.5%)	943 (18.3%)
Highest	610 (19.9%)	428 (16.7%)	659 (17.5%)	1886 (22.4%)	1536 (28.2%)	1092 (23.4%)	1172 (20.3%)
**Marital status**							
Not married	3304 (92.6%)	3050 (94.6%)	3270 (80.9%)	5486 (70.6%)	3225 (68.7%)	4000 (76.2%)	4354 (75.6%)
Married or living together	296 (7.4%)	187 (5.4%)	932 (19.1%)	2773 (29.4%)	1575 (31.3%)	1532 (23.8%)	1587 (24.4%)

SD – standard deviation

*Botswana and Zambia: 16–19.

†Wealth quintile is created using survey data on household assets, materials and durable goods; using the approach established by the Demographic and Health Surveys to measures socioeconomic status.

### Characteristics of mobile phone owners and non-owners

**Figure 1:**
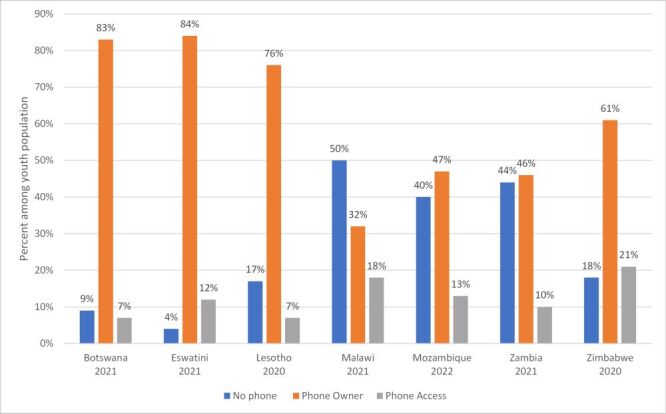
Phone ownership and access among young people (15–24) in seven Southern African countries.

Eighty-four percent of youth in Eswatini, 83% in Botswana, 76% in Lesotho, 61% in Zimbabwe, 47% in Mozambique, 47% in Zambia and 32% in Malawi were mobile phone owners *i.e*. had a personal phone (**Figure 1**). Phone access (without being a phone owner) was highest in Zimbabwe (21%) followed by Malawi (18%), Mozambique (13%), Eswatini (12%), Zambia (10%) and lowest in Lesotho and Botswana (7%). Malawi had the greatest percent of youths without phone ownership or access (50%), compared to only 4% of youth in Eswatini.

Despite 15–19-year-olds constituting more than 50% of youth in all countries, a greater percentage of older youth (20–24 years) owned cell phones than those in the younger age group (15–19 years) with pronounced differences in Zambia (youth population aged 20–24 = 51%; mobile phone owners aged 20–24 = 62%); Zimbabwe (youth population aged 20–24: 46%; mobile phone owners aged 20–24: 59%); Malawi (youth population aged 20–24: 46%; mobile phone owners aged 20–24: 62%) (**Table 2**). In the three countries where less than 50% of youth reported mobile phone ownership, the proportion of the youth population in the highest wealth quintile who owned a phone was nine times (Mozambique), five times (Malawi) and four times (Zambia) higher than the proportion in the lowest wealth quintile. Phone ownership was less common among young women compared to young men in Malawi, Mozambique and Zambia (40, 43, and 46%, respectively). Table S1–14 in the **Online Supplementary Document** present sociodemographic characteristics associated with mobile phone ownership, access and non-ownership, dis-aggregated by gender. Phone owners were more commonly urban compared to the overall youth population in Malawi (youth population: 19% urban; phone owners: 31% urban), Mozambique (youth population: 43%; phone owners: 61%) and Zambia (youth population: 43%; phone owners: 56%).

**Table 2:** Characteristics of nationally representative sample of phone owners among young in seven Southern Africa countries, 2020–2022

Variables	Botswana	Eswatini	Lesotho	Malawi	Mozambique	Zambia	Zimbabwe
	**n = 3007 (83.5%)**	**n = 3016 (83.8%)**	**n = 3216 (76.0%)**	**n = 2688 (32.1%)**	**n = 2454 (47.0%)**	**n = 2409 (46.5%)**	**n = 3565 (60.9%)**
**Age in years (SD)**	20.251 (2.412)	19.663 (2.701)	19.860 (2.693)	20.262 (2.461)	19.664 (2.768)	20.347 (2.316)	20.175 (2.448)
**Age group**							
15–19*	1186 (40.6%)	1458 (47.9%)	1406 (45.2%)	927 (37.8%)	1099 (48.8%)	912 (38.2%)	1477 (41.2%)
20–24	1821 (59.4%)	1558 (52.1%)	1810 (54.8%)	1761 (62.2%)	1355 (51.2%)	1497 (61.8%)	2088 (58.8%)
**Gender**							
Male	1307 (51.7%)	1450 (50.9%)	1249 (47.3%)	1475 (60.1%)	1259 (57.0%)	1177 (54.0%)	1520 (49.7%)
Female	1700 (48.3%)	1566 (49.1%)	1967 (52.7%)	1213 (39.9%)	1195 (43.0%)	1232 (46.0%)	2045 (50.3%)
**Residence**							
Rural	1236 (30.8%)	2463 (71.6%)	1447 (43.1%)	1806 (68.7%)	892 (38.6%)	1054 (43.7%)	2225 (59.9%)
Urban	1771 (69.2%)	553 (28.4%)	1769 (56.9%)	882 (31.3%)	1562 (61.4%)	1355 (56.3%)	1340 (40.1%)
**Highest school attended**							
No education	42 (1.8%)	18 (0.6%)	39 (1.3%)	32 (1.1%)	66 (3.2%)	31 (1.3%)	16 (0.4%)
Primary	55 (1.8%)	380 (12.0%)	564 (17.3%)	1121 (42.1%)	590 (25.5%)	353 (15.9%)	671 (17.7%)
Secondary	2373 (74.6%)	2384 (78.7%)	2301 (71.4%)	1344 (50.1%)	1688 (67.5%)	1881 (75.9%)	2647 (75.1%)
More than secondary	537 (21.8%)	234 (8.7%)	312 (9.9%)	189 (6.6%)	108 (3.7%)	144 (6.9%)	230 (6.8%)
**Wealth quintile**†							
Lowest	650 (14.9%)	640 (19.0%)	560 (15.8%)	204 (8.0%)	105 (5.1%)	297 (8.9%)	600 (14.2%)
Second	683 (21.7%)	849 (25.5%)	633 (19.6%)	394 (14.7%)	202 (9.2%)	354 (13.3%)	639 (16.8%)
Middle	545 (20.7%)	649 (21.0%)	695 (21.8%)	460 (17.0%)	264 (11.8%)	405 (18.0%)	714 (20.6%)
Fourth	581 (21.6%)	470 (17.3%)	704 (22.6%)	598 (22.7%)	659 (27.9%)	591 (24.9%)	688 (22.1%)
Highest	548 (21.0%)	408 (17.1%)	582 (20.3%)	1031 (37.6%)	1214 (46.1%)	762 (34.9%)	924 (26.4%)
**Marital status**							
Not married	2741 (92.1%)	2824 (94.6%)	2446 (79.3%)	1879 (73.1%)	1860 (76.7%)	1882 (80.8%)	2500 (72.5%)
Married	260 (7.9%)	175 (5.4%)	765 (20.7%)	807 (26.9%)	591 (23.3%)	518 (19.2%)	1061 (27.5%)

SD – standard deviation

*Botswana and Zambia: 16–19.

†Wealth quintile is created using survey data on household assets, materials and durable goods; using the approach established by the Demographic and Health Surveys to measures socioeconomic status.

**Figure 2:**
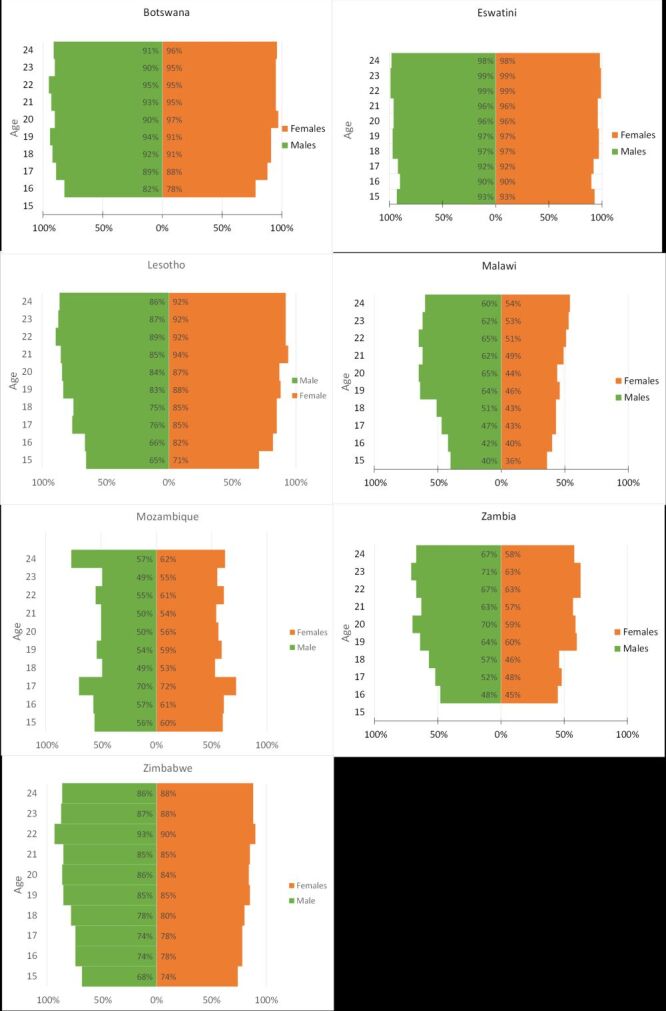
Population pyramids of mobile phone ownership by country and gender.

When dis-aggregating ownership by age and gender, four countries reach 80% ownership within an age-gender-band (**Figure 2**). Specifically, in Botswana by age 17 years (females) and 16 years (males), Eswatini by age 15 years (females and males), Lesotho age 16 years (females) age 19 years (males), and Zimbabwe age 18 years (females), 19 years (males), 80% or higher are owners. Between ages 15 and 24 years, the largest increase in mobile phone ownership is 21 percentage points for both genders in Lesotho (females aged 15 = 71% ownership, aged 24 = 92%; males aged 15 = 65% ownership, age 24 = 86%), followed by a 20 percentage point increase in Malawi among males (40 to 60%). When assessing phone ownership by one-year increments of age, both Zambia and Malawi have the largest increases 14 percentage points in Zambia among females 18 *vs*. 19 years old (46 *vs*. 60%) and in Malawi among males 18 *vs*. 19 years old (51 *vs*. 64%). Across all countries, among 15-year-olds, mobile phone ownership is the lowest in Malawi (females 36% and males 40%). While ownership increases with age in Malawi, mobile phone ownership is still the lowest among those aged 24 years (54% females, 60% males) compared to other countries.

The odds of mobile phone ownership were independently and significantly associated with older age across all countries: 20–24-year-olds were 1.3 to 2.8 times more likely to own phones than 15–19-year-olds (**Table 3**). Mobile phone ownership was also significantly associated with higher education: in all countries, those with secondary education or higher had 1.4 to 3.3 times higher odds of mobile phone ownership than those with less education. Women were significantly less likely to own phones than men in Malawi (odds ratio (OR) = 0.7; 95% confidence interval (CI) = 0.6–0.8), Mozambique (OR = 0.6; 95% CI = 0.5–0.7), and Zambia (OR = 0.8; 95% CI = 0.7–0.9). In all countries except Eswatini, odds of phone ownership were higher amongst those in the four higher wealth quintile, compared to the lowest wealth quintile.

**Table 3:** Odds of mobile phone ownership by country and select sociodemographic characteristics

Variables	Botswana		Eswatini		Lesotho		Malawi		Mozambique		Zambia		Zimbabwe	
	**OR**	**95% CI (lower–upper)**	**OR**	**95% CI (lower–upper)**	**OR**	**95% CI (lower–upper)**	**OR**	**95% CI (lower–upper)**	**OR**	**95% CI (lower–upper)**	**OR**	95% CI (lower–upper)	OR	95% CI (lower–upper)
Age, 20–24 *vs*. 15–19* (Ref)	2.3	1.7–3.1	2.8	1.9–4.3	2.1	1.7–2.5	1.5	1.4–1.7	1.3	1.1–1.5	1.8	1.6–2.1	1.8	1.6–2.1
Gender, female *vs*. male (Ref)	1.1	0.8–1.5	1.0	0.7–1.4	1.5	1.3–1.8	0.7	0.6–0.8	0.6	0.5–0.7	0.8	0.7–0.9	1.0	0.9–1.2
Geography, urban *vs*. rural (Ref)	1.1	0.8–1.5	1.4	0.8–2.6	1.3	1.0–1.6	1.2	1–1.4	1.1	0.9–1.4	1.0	0.8–1.3	0.9	0.7–1.3
Education, secondary and more *vs*. less than secondary (Ref)	2.8	1.7–4.6	1.4	0.9–2.2	2.1	1.8–2.6	2.7	2.4–3	3.3	2.7–3.9	2.4	2–2.8	2	1.7–2.4
Wealth quintiles, lowest (Ref)	-	-	-	-	-	-	-	-	-	-	-	-	-	-
*Second*	1.8	1.2–2.7	1.1	0.7–1.7	1.5	1.2–1.9	2	1.7–2.3	1.9	1.4–2.5	2	1.6–2.5	1.5	1.3–1.9
*Middle*	2.1	1.3–3.5	1.1	0.7–1.8	1.9	1.4–2.5	2.2	1.8–2.6	2.6	1.9–3.4	3.3	2.6–4.2	2	1.6–2.5
*Fourth*	2.6	1.6–4.2	1.3	0.7–2.4	3	2.1–4.2	2.7	2.3–3.2	4.5	3.3–6.1	4.2	3.2–5.6	2.7	1.9–3.8
*Highest*	2.3	1.4–4	1.1	0.6–2.1	3.5	2.3–5.3	4.6	3.8–5.6	18.1	12.4–26.4	7.7	5.7–10.4	3.5	2.3–5.4

CI – confidence interval, OR – odds ratio

*Botswana and Zambia: 16–19.

## DISCUSSION

Despite differing levels of mobile phone ownership by country, with over 80% of youth in Eswatini and Botswana owning a phone compared to less than 50% in Malawi, Mozambique and Zambia, some characteristics of phone owners were consistent across countries. By analysing nationally representative data on young adults ages 15–24 years, we found that being over age 19 years and completing secondary school or higher was associated with mobile phone ownership across all countries. In six of seven countries, higher household wealth quintile was associated with higher odds of phone ownership among individual young people. In four countries, 80% ownership was attained by at least age 24 years amongst both genders (Botswana, Eswatini, Lesotho, Zimbabwe). The three countries with ownership below 50% are lowest on the development index out of all study countries and showed the greatest inequities – particularly gender and wealth – in ownership. Inequities in ownership by sociodemographics are unlikely to significantly reduce any time soon given the compound annual growth rate of mobile phone ownership has slowed significantly [20].

The existent research on mobile phone ownership among young adults in SSA is minimal and what does exist is not nationally representative [9]. The demographic profile of phone owners we identified (being over 18, educated, male and wealthy) was consistent with previous studies that focus on adults [21]. In the past ten years, research from Mozambique [11], South Africa, Ghana, Uganda, Nigeria, Kenya, and Tanzania [3] identified phone owners with similar demographic profiles to the adolescents we studied. In the past five years, research among women in SSA, particularly Burkina Faso [13], Nigeria [22], and Tanzania [23] have found phone ownership is associated with better health outcomes. The social significance of having a mobile phone is profound in SSA, with one study finding that women who own a mobile phone are more informed about sexual and reproductive health, are more empowered to make independent decisions [16], and have lower total fertility rates [15]. Thus, mHealth programmes are more likely to reach those who are in better health rather than reaching those with the most need.

Although myriad studies have examined the use of mHealth for adolescents, we are among the first to analyse which youth could be reached by the aforementioned mHealth programmes. By focusing on the fastest growing population of phone owners in SSA, we examine the population that will be driving phone ownership for years to come. Our findings show the variability in mobile phone ownership rates in seven countries, helping mHealth programmes identify whom they could reach via phone.

A main strength of this study is the use of nationally representative, population-based data on adolescents and young adults. As such, the results provide a more representative view of ownership among youth than previously presented. Furthermore, having data on youth ages 15–17 allows us to understand ownership among teens during influential years. Another strength is that PHIA survey questionnaires ask all participants about mobile phone access and ownership, thereby helping the respondent differentiate between the two. Previous analyses of phone ownership [13] have not had as clear of a distinction, which may have caused over-reporting of mobile phone ownership (measurement error).

However, the interviewer first asked if we could attempt to contact the respondent by phone, which may have resulted in under-reporting of ownership and access for those who did not want to continue study participation via phone (*i.e*. we did not directly ask ‘do you own or have access to a phone?’). While we disaggregated ownership by gender and presented results in supplemental tables, we did not conduct multivariable analyses by gender due to sample size limitations in countries with low female ownership. Previous analyses have shown that the sociodemographic characteristics of ownership differ by gender and it is probable that female phone owners are particularly different than female non-owners for youth in countries where barriers including financial (most frequently reported reason for non-ownership among African adult women in 2020 [12]), literacy and skills and gender norms would inhibit young women from mobile phone ownership. The implications of mobile phone ownership on women’s lives – from empowering livelihoods to better health outcomes are addressed in the existing literature [24]. The generalisability of the results outside of Southern Africa may be limited particularly because Southern African countries have higher phone ownership than other areas of SSA and higher smart phone ownership [20], which could impact ownership patterns. Specifically, inequities may be less profound in Southern Africa compared to other parts of the continent. Finally, we do not have data on type (basic or smart) or frequency of use of mobile phones. As shown among women in Tanzania [23] and adolescents in Zimbabwe [25], mobile phone ownership does not translate to use. Understanding how often youth charge and use their mobile phone and for what purposes (such as sending messages, making calls or using the internet) could be added to surveys to understand to what extent youth who own phones are likely to be reached. A recent publication from Zimbabwe highlights questions about ownership and phone use that would help understand phone use, rather than just ownership [25].

## CONCLUSIONS

Our study informs policy makers about who they can anticipate reaching via a mobile phone-based data collection or mHealth programme. Considering the consistent association between key sociodemographic characteristics and phone ownership across countries, those employing mobile phones for youth health programmes may not reach the general youth population.

## Additional material


Online Supplementary Document
