# Supplementary material for: Mobile phone ownership among young adults in seven Southern African countries
Source: J Glob Health. 2025 Apr 18;15:04123. doi: 10.7189/jogh.15.04123 (PMC12006829; doi:10.7189/jogh.15.04123)
Supplement: Online Supplementary Document [file jogh-15-04123-s001.pdf]

Supplement to: Greenleaf AR, Millington M, Robles-Torres L, Asiimwe F, Diakabana H, Francis SD, Mharadze T, Justman J. Mobile phone ownership among young adults in seven Southern African countries. J Glob Health. 2025;15:04123.

Supplemental Table 1. BAIS2021 (Botswana) FEMALE Phone ownership, access and non-ownership characteristics

	No phone	Phone owner	Phone Access	Total	p-value
**N**	(N=160) (9.0%)	(N=1,700) (83.4%)	(N=152) (7.6%)	(N=2,012) (100.0%)	
**Age in years**	18.623 (2.381)	20.376 (2.414)	17.314 (1.826)	19.985 (2.546)	<0.001
**Gender**					
Female	160 (100.0%)	1,700 (100.0%)	152 (100.0%)	2,012 (100.0%)	
**Residence**					
Rural	69 (31.7%)	703 (31.0%)	63 (33.0%)	835 (31.2%)	0.917
Urban	91 (68.3%)	997 (69.0%)	89 (67.0%)	1,177 (68.8%)	
**Highest school attended**					
No education	4 (4.1%)	20 (1.2%)	4 (2.7%)	28 (1.6%)	<0.001
Primary	12 (2.8%)	26 (1.5%)	12 (7.2%)	50 (2.0%)	
Secondary	127 (75.8%)	1,318 (73.1%)	131 (87.2%)	1,576 (74.4%)	
More than secondary	17 (17.3%)	336 (24.1%)	5 (2.8%)	358 (21.9%)	
**Wealth quintiles**					
Lowest	58 (23.5%)	375 (15.1%)	53 (27.8%)	486 (16.8%)	0.004
Second	39 (21.9%)	382 (21.8%)	43 (28.3%)	464 (22.3%)	
Middle	16 (19.0%)	309 (20.0%)	21 (13.4%)	346 (19.4%)	
Fourth	23 (12.6%)	324 (22.8%)	24 (18.9%)	371 (21.6%)	
Highest	24 (23.0%)	310 (20.3%)	11 (11.6%)	345 (19.9%)	
**Marital status**					
Not married	146 (93.2%)	1,499 (88.6%)	138 (93.8%)	1,783 (89.4%)	0.038
Married	14 (6.8%)	198 (11.4%)	14 (6.2%)	226 (10.6%)	

Mean (Standard deviation): p-value from linear regression.

Frequency (Percent%): p-value from Pearson test.

Statistics computed using the survey weights.

Tests adjusted for the survey design.

**Age group**					
15-19	107 (70.9%)	631 (39.0%)	122 (88.0%)	860 (45.6%)	<0.001
20-24	53 (29.1%)	1,069 (61.0%)	30 (12.0%)	1,152 (54.4%)	

Mean (Standard deviation): p-value from linear regression.

Frequency (Percent%): p-value from Pearson test.

Statistics computed using the survey weights.

Tests adjusted for the survey design.

Supplemental Table 2. BAIS2021 (Botswana) MALE Phone ownership, access and non-ownership characteristics

	No phone	Phone owner	Phone Access	Total	p-value
**N**	(N=171) (9.6%)	(N=1,307) (83.6%)	(N=117) (6.8%)	(N=1,595) (100.0%)	
**Age in years**	19.175 (2.859)	20.134 (2.401)	17.426 (2.071)	19.857 (2.529)	<0.001
**Gender**					
Male	171 (100.0%)	1,307 (100.0%)	117 (100.0%)	1,595 (100.0%)	
**Residence**					
Rural	90 (44.0%)	533 (30.7%)	59 (42.0%)	682 (32.7%)	0.009
Urban	81 (56.0%)	774 (69.3%)	58 (58.0%)	913 (67.3%)	
**Highest school attended**					
No education	8 (4.8%)	22 (2.3%)	2 (1.1%)	32 (2.5%)	0.003
Primary	22 (10.4%)	29 (2.0%)	11 (8.7%)	62 (3.3%)	
Secondary	130 (75.6%)	1,055 (76.0%)	99 (80.2%)	1,284 (76.2%)	
More than secondary	11 (9.2%)	201 (19.7%)	5 (10.0%)	217 (18.0%)	
**Wealth quintiles**					
Lowest	75 (32.8%)	275 (14.8%)	40 (24.5%)	390 (17.2%)	0.001
Second	35 (22.0%)	301 (21.7%)	27 (24.1%)	363 (21.9%)	
Middle	20 (16.1%)	236 (21.3%)	18 (20.6%)	274 (20.7%)	
Fourth	23 (18.4%)	257 (20.5%)	23 (19.3%)	303 (20.2%)	
Highest	18 (10.5%)	238 (21.7%)	9 (11.4%)	265 (20.0%)	
**Marital status**					
Not married	167 (98.2%)	1,242 (95.4%)	112 (96.1%)	1,521 (95.7%)	0.130
Married	4 (1.8%)	62 (4.6%)	4 (3.9%)	70 (4.3%)	
**Age group**					
15-19	95 (55.3%)	555 (42.0%)	98 (83.0%)	748 (46.0%)	<0.001
20-24	76 (44.7%)	752 (58.0%)	19 (17.0%)	847 (54.0%)	

Mean (Standard deviation): p-value from linear regression.

Frequency (Percent%): p-value from Pearson test.

Statistics computed using the survey weights.

Tests adjusted for the survey design.

Supplemental Table 3. SHIMS32021 (Eswatini) FEMALE Phone ownershiop, access and non-ownership characteristics

	No phone	Phone owner	Phone Access	Total	p-value
**N**	(N=78) (4.1%)	(N=1,566) (83.0%)	(N=258) (12.9%)	(N=1,902) (100.0%)	
**Age in years**	17.314 (2.579)	19.721 (2.681)	17.031 (2.381)	19.276 (2.821)	<0.001
**Gender**					
Female	78 (100.0%)	1,566 (100.0%)	258 (100.0%)	1,902 (100.0%)	
**Residence**					
Rural	70 (81.8%)	1,258 (69.1%)	238 (86.8%)	1,566 (71.9%)	<0.001
Urban	8 (18.2%)	308 (30.9%)	20 (13.2%)	336 (28.1%)	
**Highest school attended**					
No education	1 (1.1%)	9 (0.6%)	2 (0.7%)	12 (0.6%)	<0.001
Primary	9 (12.2%)	151 (9.4%)	83 (31.8%)	243 (12.4%)	
Secondary	66 (82.7%)	1,272 (80.2%)	169 (65.4%)	1,507 (78.4%)	
More than secondary	2 (4.0%)	134 (9.9%)	4 (2.1%)	140 (8.6%)	
**Wealth quintiles**					
Lowest	21 (24.8%)	345 (19.4%)	91 (33.1%)	457 (21.4%)	<0.001
Second	27 (32.9%)	436 (24.6%)	75 (27.9%)	538 (25.3%)	
Middle	14 (17.6%)	307 (19.0%)	53 (20.1%)	374 (19.0%)	
Fourth	10 (14.4%)	259 (19.0%)	26 (11.5%)	295 (17.9%)	
Highest	6 (10.4%)	219 (18.1%)	13 (7.4%)	238 (16.4%)	
**Marital status**					
Not married	76 (99.0%)	1,404 (90.9%)	243 (95.1%)	1,723 (91.8%)	<0.001
Married	1 (1.0%)	151 (9.1%)	13 (4.9%)	165 (8.2%)	
**Age group**					
15-19	65 (80.2%)	723 (46.9%)	218 (85.5%)	1,006 (53.2%)	<0.001
20-24	13 (19.8%)	843 (53.1%)	40 (14.5%)	896 (46.8%)	

Mean (Standard deviation): p-value from linear regression.

Frequency (Percent%): p-value from Pearson test.

Statistics computed using the survey weights.

Tests adjusted for the survey design.

Supplemental Table 4. SHIMS32021 (Eswatini) MALE Phone ownershiop, access and non-ownership characteristics

	No phone	Phone owner	Phone Access	Total	p-value
**N**	(N=82) (4.5%)	(N=1,450) (84.6%)	(N=200) (10.9%)	(N=1,732) (100.0%)	
**Age in years**	17.687 (2.549)	19.608 (2.720)	17.021 (2.504)	19.240 (2.830)	<0.001
**Gender**					
Male	82 (100.0%)	1,450 (100.0%)	200 (100.0%)	1,732 (100.0%)	
**Residence**					
Rural	74 (84.9%)	1,205 (73.9%)	184 (86.0%)	1,463 (75.7%)	<0.001
Urban	8 (15.1%)	245 (26.1%)	16 (14.0%)	269 (24.3%)	
**Highest school attended**					
No education	1 (2.4%)	9 (0.6%)	0 (0.0%)	10 (0.6%)	<0.001
Primary	29 (35.3%)	229 (14.6%)	108 (55.1%)	366 (19.9%)	
Secondary	50 (60.2%)	1,112 (77.3%)	92 (44.9%)	1,254 (73.0%)	
More than secondary	2 (2.2%)	100 (7.6%)	0 (0.0%)	102 (6.5%)	
**Wealth quintiles**					
Lowest	26 (28.8%)	295 (18.7%)	78 (36.4%)	399 (21.1%)	<0.001
Second	20 (23.6%)	413 (26.4%)	64 (31.4%)	497 (26.9%)	
Middle	19 (22.0%)	342 (23.1%)	34 (16.9%)	395 (22.4%)	
Fourth	7 (10.4%)	211 (15.7%)	15 (8.7%)	233 (14.7%)	
Highest	10 (15.1%)	189 (16.1%)	9 (6.6%)	208 (15.0%)	
**Marital status**					
Not married	81 (100.0%)	1,420 (98.0%)	196 (100.0%)	1,697 (98.3%)	<0.001
Married	0 (0.0%)	24 (2.0%)	0 (0.0%)	24 (1.7%)	
**Age group**					
15-19	63 (75.2%)	735 (48.9%)	170 (84.1%)	968 (53.9%)	<0.001
20-24	19 (24.8%)	715 (51.1%)	30 (15.9%)	764 (46.1%)	

Mean (Standard deviation): p-value from linear regression.

Frequency (Percent%): p-value from Pearson test.

Statistics computed using the survey weights.

Tests adjusted for the survey design.

Supplemental Table 5. LePHIA 2020 (Lesotho) FEMALE Phone ownership, access and non-ownership characteristics

	No phone	Phone owner	Phone Access	Total	p-value
**N**	(N=329) (12.9%)	(N=1,967) (80.1%)	(N=171) (7.0%)	(N=2,467) (100.0%)	
**Age in years**	18.410 (2.800)	19.863 (2.675)	17.685 (2.589)	19.523 (2.775)	<0.001
**Gender**					
Female	329 (100.0%)	1,967 (100.0%)	171 (100.0%)	2,467 (100.0%)	
**Residence**					
Rural	222 (66.1%)	900 (43.2%)	87 (48.4%)	1,209 (46.5%)	<0.001
Urban	107 (33.9%)	1,067 (56.8%)	84 (51.6%)	1,258 (53.5%)	
**Highest school attended**					
No education	5 (1.5%)	19 (0.9%)	3 (1.9%)	27 (1.0%)	<0.001
Primary	114 (33.0%)	281 (13.3%)	44 (25.1%)	439 (16.7%)	
Secondary	196 (61.1%)	1,477 (75.7%)	124 (73.0%)	1,797 (73.6%)	
More than secondary	14 (4.4%)	190 (10.1%)	0 (0.0%)	204 (8.7%)	
**Wealth quintiles**					
Lowest	146 (41.8%)	373 (17.2%)	45 (24.3%)	564 (20.9%)	<0.001
Second	68 (21.3%)	371 (18.6%)	43 (25.0%)	482 (19.4%)	
Middle	57 (17.9%)	411 (21.1%)	30 (18.2%)	498 (20.5%)	
Fourth	38 (12.6%)	436 (23.2%)	34 (21.3%)	508 (21.7%)	
Highest	19 (6.3%)	350 (19.9%)	16 (11.1%)	385 (17.5%)	
**Marital status**					
Not married	235 (73.3%)	1,314 (68.8%)	127 (76.9%)	1,676 (70.0%)	0.023
Married	94 (26.7%)	649 (31.2%)	43 (23.1%)	786 (30.0%)	
**Age group**					
15-19	212 (66.1%)	858 (45.3%)	127 (76.4%)	1,197 (50.2%)	<0.001
20-24	117 (33.9%)	1,109 (54.7%)	44 (23.6%)	1,270 (49.8%)	

Mean (Standard deviation): p-value from linear regression.

Frequency (Percent%): p-value from Pearson test.

Statistics computed using the survey weights.

Tests adjusted for the survey design.

Supplemental Table 6. LePHIA 2020 (Lesotho) MALE Phone ownership, access and non-ownership characteristics

	No phone	Phone owner	Phone Access	Total	p-value
**N**	(N=364) (20.4%)	(N=1,249) (71.9%)	(N=131) (7.7%)	(N=1,744) (100.0%)	
**Age in years**	18.415 (2.789)	19.857 (2.685)	18.109 (2.677)	19.428 (2.792)	<0.001
**Gender**					
Male	364 (100.0%)	1,249 (100.0%)	131 (100.0%)	1,744 (100.0%)	
**Residence**					
Rural	258 (70.5%)	547 (42.9%)	73 (54.4%)	878 (49.4%)	<0.001
Urban	106 (29.5%)	702 (57.1%)	58 (45.6%)	866 (50.6%)	
**Highest school attended**					
No education	22 (6.0%)	20 (1.7%)	2 (1.1%)	44 (2.5%)	<0.001
Primary	173 (47.2%)	283 (21.8%)	51 (39.5%)	507 (28.3%)	
Secondary	163 (45.2%)	824 (66.7%)	75 (57.4%)	1,062 (61.6%)	
More than secondary	6 (1.6%)	122 (9.8%)	3 (2.1%)	131 (7.5%)	
**Wealth quintiles**					
Lowest	140 (37.1%)	187 (14.1%)	38 (27.5%)	365 (19.9%)	<0.001
Second	103 (28.9%)	262 (20.7%)	28 (21.9%)	393 (22.5%)	
Middle	61 (17.8%)	284 (22.6%)	21 (16.3%)	366 (21.2%)	
Fourth	30 (8.4%)	268 (21.9%)	25 (20.4%)	323 (19.0%)	
Highest	25 (7.8%)	232 (20.7%)	17 (13.8%)	274 (17.5%)	
**Marital status**					
Not married	338 (93.6%)	1,132 (90.9%)	124 (96.2%)	1,594 (91.9%)	0.007
Married	24 (6.4%)	116 (9.1%)	6 (3.8%)	146 (8.1%)	
**Age group**					
15-19	238 (67.5%)	548 (45.1%)	92 (72.3%)	878 (51.7%)	<0.001
20-24	126 (32.5%)	701 (54.9%)	39 (27.7%)	866 (48.3%)	

Mean (Standard deviation): p-value from linear regression.

Frequency (Percent%): p-value from Pearson test.

Statistics computed using the survey weights.

Tests adjusted for the survey design.

Supplemental Table 7. MPHIA 2020 (Malawi) FEMALE Phone ownership, access and non-ownership characteristics

	No phone	Phone owner	Phone Access	Total	p-value
**N**	(N=2,474) (54.8%)	(N=1,213) (24.9%)	(N=888) (20.3%)	(N=4,575) (100.0%)	
**Age in years**	18.958 (2.731)	20.351 (2.487)	18.522 (2.756)	19.216 (2.768)	<0.001
**Gender**					
Female	2,474 (100.0%)	1,213 (100.0%)	888 (100.0%)	4,575 (100.0%)	
**Residence**					
Rural	2,197 (89.3%)	764 (64.2%)	702 (80.0%)	3,663 (81.2%)	<0.001
Urban	277 (10.7%)	449 (35.8%)	186 (20.0%)	912 (18.8%)	
**Highest school attended**					
No education	118 (4.7%)	14 (1.1%)	10 (1.1%)	142 (3.1%)	<0.001
Primary	1,925 (77.9%)	520 (42.3%)	576 (65.3%)	3,021 (66.4%)	
Secondary	425 (17.3%)	586 (49.3%)	299 (33.4%)	1,310 (28.5%)	
More than secondary	6 (0.2%)	93 (7.3%)	2 (0.3%)	101 (2.0%)	
**Wealth quintiles**					
Lowest	681 (27.8%)	77 (6.4%)	114 (13.0%)	872 (19.5%)	<0.001
Second	546 (21.7%)	169 (13.8%)	183 (19.6%)	898 (19.3%)	
Middle	506 (20.4%)	196 (15.8%)	166 (18.9%)	868 (19.0%)	
Fourth	469 (18.9%)	243 (20.7%)	209 (23.8%)	921 (20.4%)	
Highest	272 (11.1%)	528 (43.3%)	216 (24.6%)	1,016 (21.9%)	
**Marital status**					
Not married	1,226 (54.9%)	716 (62.5%)	526 (65.7%)	2,468 (59.0%)	<0.001
Married	1,246 (45.1%)	497 (37.5%)	361 (34.3%)	2,104 (41.0%)	
**Age group**					
15-19	1,227 (57.3%)	374 (36.8%)	498 (64.6%)	2,099 (53.7%)	<0.001
20-24	1,247 (42.7%)	839 (63.2%)	390 (35.4%)	2,476 (46.3%)	

Mean (Standard deviation): p-value from linear regression.

Frequency (Percent%): p-value from Pearson test.

Statistics computed using the survey weights.

Tests adjusted for the survey design.

Supplemental Table 8. MPHIA 2020 (Malawi) MALE Phone ownership, access and non-ownership characteristics

	No phone	Phone owner	Phone Access	Total	p-value
**N**	(N=1,641) (44.9%)	(N=1,475) (39.7%)	(N=575) (15.5%)	(N=3,691) (100.0%)	
**Age in years**	18.701 (2.807)	20.203 (2.475)	18.032 (2.524)	19.193 (2.772)	<0.001
**Gender**					
Male	1,641 (100.0%)	1,475 (100.0%)	575 (100.0%)	3,691 (100.0%)	
**Residence**					
Rural	1,421 (87.6%)	1,042 (71.6%)	449 (80.2%)	2,912 (80.2%)	<0.001
Urban	220 (12.4%)	433 (28.4%)	126 (19.8%)	779 (19.8%)	
**Highest school attended**					
No education	55 (3.4%)	18 (1.2%)	3 (0.5%)	76 (2.0%)	<0.001
Primary	1,240 (76.2%)	601 (42.0%)	396 (69.6%)	2,237 (61.6%)	
Secondary	337 (20.0%)	758 (50.7%)	172 (29.1%)	1,267 (33.5%)	
More than secondary	8 (0.5%)	96 (6.2%)	4 (0.9%)	108 (2.8%)	
**Wealth quintiles**					
Lowest	358 (22.7%)	127 (9.0%)	58 (10.2%)	543 (15.3%)	<0.001
Second	348 (21.0%)	225 (15.3%)	98 (17.3%)	671 (18.1%)	
Middle	360 (21.8%)	264 (17.9%)	122 (21.2%)	746 (20.1%)	
Fourth	357 (21.8%)	355 (24.0%)	148 (26.8%)	860 (23.5%)	
Highest	218 (12.8%)	503 (33.8%)	149 (24.5%)	870 (22.9%)	
**Marital status**					
Not married	1,335 (82.5%)	1,163 (80.2%)	520 (91.3%)	3,018 (83.0%)	<0.001
Married	306 (17.5%)	310 (19.8%)	53 (8.7%)	669 (17.0%)	
**Age group**					
15-19	1,003 (62.4%)	553 (38.4%)	415 (73.3%)	1,971 (54.6%)	<0.001
20-24	638 (37.6%)	922 (61.6%)	160 (26.7%)	1,720 (45.4%)	

Mean (Standard deviation): p-value from linear regression.

Frequency (Percent%): p-value from Pearson test.

Statistics computed using the survey weights.

Tests adjusted for the survey design.

Supplemental Table 9. INSIDA2021 (Mozambique) FEMALE Phone ownership, access and non-ownership characteristics

	No phone	Phone owner	Phone Access	Total	p-value
**N**	(N=1,104) (46.0%)	(N=1,195) (39.6%)	(N=372) (14.4%)	(N=2,671) (100.0%)	
**Age in years**	19.407 (2.474)	19.535 (2.777)	18.321 (2.654)	19.302 (2.655)	<0.001
**Gender**					
Female	1,104 (100.0%)	1,195 (100.0%)	372 (100.0%)	2,671 (100.0%)	
**Residence**					
Rural	822 (74.6%)	405 (36.7%)	183 (51.3%)	1,410 (56.3%)	<0.001
Urban	282 (25.4%)	790 (63.3%)	189 (48.7%)	1,261 (43.7%)	
**Highest school attended**					
No education	240 (23.0%)	31 (3.1%)	22 (6.2%)	293 (12.7%)	<0.001
Primary	648 (58.7%)	251 (22.4%)	165 (44.8%)	1,064 (42.3%)	
Secondary	213 (18.2%)	856 (70.2%)	185 (49.0%)	1,254 (43.3%)	
More than secondary	1 (0.1%)	55 (4.2%)	0 (0.0%)	56 (1.7%)	
**Wealth quintiles**					
Lowest	281 (26.7%)	36 (3.3%)	20 (5.8%)	337 (14.4%)	<0.001
Second	286 (24.8%)	65 (6.0%)	51 (14.7%)	402 (15.9%)	
Middle	249 (21.3%)	116 (10.9%)	66 (20.2%)	431 (17.0%)	
Fourth	208 (20.4%)	348 (30.9%)	109 (28.5%)	665 (25.7%)	
Highest	76 (6.9%)	626 (48.9%)	125 (30.7%)	827 (26.9%)	
**Marital status**					
Not married	440 (43.0%)	839 (70.9%)	238 (64.9%)	1,517 (57.2%)	<0.001
Married	664 (57.0%)	354 (29.1%)	134 (35.1%)	1,152 (42.8%)	
**Age group**					
15-19	501 (51.2%)	545 (50.4%)	233 (67.3%)	1,279 (53.2%)	<0.001
20-24	603 (48.8%)	650 (49.6%)	139 (32.7%)	1,392 (46.8%)	

Mean (Standard deviation): p-value from linear regression.

Frequency (Percent%): p-value from Pearson test.

Statistics computed using the survey weights.

Tests adjusted for the survey design.

Supplemental Table 10. INSIDA2021 (Mozambique) MALE Phone ownership, access and non-ownership characteristics

	No phone	Phone owner	Phone Access	Total	p-value
**N**	(N=665) (34.5%)	(N=1,259) (54.8%)	(N=208) (10.7%)	(N=2,132) (100.0%)	
**Age in years**	18.986 (2.538)	19.760 (2.788)	17.175 (1.971)	19.217 (2.738)	<0.001
**Gender**					
Male	665 (100.0%)	1,259 (100.0%)	208 (100.0%)	2,132 (100.0%)	
**Residence**					
Rural	531 (80.0%)	487 (40.1%)	90 (48.8%)	1,108 (54.8%)	<0.001
Urban	134 (20.0%)	772 (59.9%)	118 (51.2%)	1,024 (45.2%)	
**Highest school attended**					
No education	88 (14.7%)	35 (3.2%)	11 (5.7%)	134 (7.5%)	<0.001
Primary	402 (59.7%)	339 (27.9%)	82 (43.7%)	823 (40.5%)	
Secondary	174 (25.5%)	832 (65.5%)	114 (50.4%)	1,120 (50.1%)	
More than secondary	1 (0.1%)	53 (3.4%)	1 (0.2%)	55 (1.9%)	
**Wealth quintiles**					
Lowest	169 (26.3%)	69 (6.4%)	14 (6.6%)	252 (13.3%)	<0.001
Second	181 (27.9%)	137 (11.6%)	18 (14.6%)	336 (17.5%)	
Middle	156 (22.0%)	148 (12.4%)	30 (14.7%)	334 (16.0%)	
Fourth	126 (19.3%)	311 (25.6%)	54 (27.1%)	491 (23.6%)	
Highest	29 (4.5%)	588 (44.0%)	92 (37.0%)	709 (29.6%)	
**Marital status**					
Not married	492 (75.8%)	1,021 (81.1%)	195 (93.9%)	1,708 (80.7%)	<0.001
Married	173 (24.2%)	237 (18.9%)	13 (6.1%)	423 (19.3%)	
**Age group**					
15-19	353 (58.6%)	554 (47.7%)	174 (86.1%)	1,081 (55.6%)	<0.001
20-24	312 (41.4%)	705 (52.3%)	34 (13.9%)	1,051 (44.4%)	

Mean (Standard deviation): p-value from linear regression.

Frequency (Percent%): p-value from Pearson test.

Statistics computed using the survey weights.

Tests adjusted for the survey design.

Supplemental Table 11. ZAMPHIA2021 (Zimbabwe) FEMALE Phone ownership, access and non-ownership characteristics

	No phone	Phone owner	Phone Access	Total	p-value
**N**	(N=1,587) (46.6%)	(N=1,232) (42.1%)	(N=315) (11.3%)	(N=3,134) (100.0%)	
**Age in years**	19.427 (2.713)	20.446 (2.273)	18.293 (2.367)	19.729 (2.588)	<0.001
**Gender**					
Female	1,587 (100.0%)	1,232 (100.0%)	315 (100.0%)	3,134 (100.0%)	
**Residence**					
Rural	1,215 (74.4%)	472 (38.2%)	181 (54.3%)	1,868 (56.9%)	<0.001
Urban	372 (25.6%)	760 (61.8%)	134 (45.7%)	1,266 (43.1%)	
**Highest school attended**					
No education	117 (7.2%)	12 (1.0%)	11 (4.6%)	140 (4.3%)	<0.001
Primary	727 (43.1%)	153 (13.7%)	116 (33.6%)	996 (29.7%)	
Secondary	735 (49.1%)	991 (78.8%)	187 (61.3%)	1,913 (63.0%)	
More than secondary	8 (0.6%)	76 (6.5%)	1 (0.5%)	85 (3.1%)	
**Wealth quintiles**					
Lowest	659 (33.6%)	139 (8.1%)	57 (11.0%)	855 (20.3%)	<0.001
Second	387 (23.4%)	146 (10.3%)	72 (20.4%)	605 (17.5%)	
Middle	242 (19.4%)	193 (16.9%)	53 (19.8%)	488 (18.4%)	
Fourth	186 (15.0%)	319 (26.8%)	69 (25.0%)	574 (21.1%)	
Highest	113 (8.7%)	435 (37.9%)	64 (23.8%)	612 (22.7%)	
**Marital status**					
Not married	844 (56.3%)	896 (74.2%)	206 (68.5%)	1,946 (65.2%)	<0.001
Married	736 (43.7%)	333 (25.8%)	109 (31.5%)	1,178 (34.8%)	
**Age group**					
15-19	777 (54.4%)	430 (37.2%)	205 (70.2%)	1,412 (48.9%)	<0.001
20-24	810 (45.6%)	802 (62.8%)	110 (29.8%)	1,722 (51.1%)	

Mean (Standard deviation): p-value from linear regression.

Frequency (Percent%): p-value from Pearson test.

Statistics computed using the survey weights.

Tests adjusted for the survey design.

Supplemental Table 12. ZAMPHIA2021 (Zimbabwe) MALE Phone ownership, access and non-ownership characteristics

	No phone	Phone owner	Phone Access	Total	p-value
**N**	(N=1,064) (40.6%)	(N=1,177) (51.0%)	(N=177) (8.4%)	(N=2,418) (100.0%)	
**Age in years**	19.242 (2.694)	20.263 (2.375)	18.280 (2.230)	19.682 (2.577)	<0.001
**Gender**					
Male	1,064 (100.0%)	1,177 (100.0%)	177 (100.0%)	2,418 (100.0%)	
**Residence**					
Rural	787 (69.5%)	582 (48.3%)	84 (54.3%)	1,453 (57.4%)	<0.001
Urban	277 (30.5%)	595 (51.7%)	93 (45.7%)	965 (42.6%)	
**Highest school attended**					
No education	62 (5.6%)	19 (1.6%)	3 (0.9%)	84 (3.2%)	<0.001
Primary	470 (43.5%)	200 (17.7%)	59 (34.4%)	729 (29.6%)	
Secondary	518 (49.3%)	890 (73.4%)	113 (61.4%)	1,521 (62.6%)	
More than secondary	12 (1.5%)	68 (7.3%)	2 (3.2%)	82 (4.6%)	
**Wealth quintiles**					
Lowest	342 (24.9%)	158 (9.5%)	16 (6.2%)	516 (15.5%)	<0.001
Second	282 (23.6%)	208 (15.9%)	24 (10.1%)	514 (18.6%)	
Middle	181 (19.4%)	212 (18.9%)	43 (27.5%)	436 (19.8%)	
Fourth	154 (18.8%)	272 (23.4%)	46 (28.4%)	472 (21.9%)	
Highest	105 (13.2%)	327 (32.3%)	48 (27.8%)	480 (24.2%)	
**Marital status**					
Not married	894 (86.5%)	986 (86.4%)	174 (98.3%)	2,054 (87.5%)	<0.001
Married	166 (13.5%)	185 (13.6%)	3 (1.7%)	354 (12.5%)	
**Age group**					
15-19	609 (57.3%)	482 (39.1%)	137 (72.0%)	1,228 (49.2%)	<0.001
20-24	455 (42.7%)	695 (60.9%)	40 (28.0%)	1,190 (50.8%)	

Mean (Standard deviation): p-value from linear regression.

Frequency (Percent%): p-value from Pearson test.

Statistics computed using the survey weights.

Tests adjusted for the survey design.

Supplemental Table 13. ZIMPHIA2020 (Zimbabwe) FEMALE Phone ownership, access and non-ownership characteristics

	No phone	Phone owner	Phone Access	Total	p-value
**N**	(N=588) (17.2%)	(N=2,045) (60.8%)	(N=734) (22.0%)	(N=3,367) (100.0%)	
**Age in years**	18.635 (2.659)	20.126 (2.449)	17.776 (2.569)	19.353 (2.704)	<0.001
**Gender**					
Female	588 (100.0%)	2,045 (100.0%)	734 (100.0%)	3,367 (100.0%)	
**Residence**					
Rural	478 (77.6%)	1,192 (54.9%)	574 (75.3%)	2,244 (63.3%)	<0.001
Urban	110 (22.4%)	853 (45.1%)	160 (24.7%)	1,123 (36.7%)	
**Highest school attended**					
No education	10 (2.1%)	10 (0.4%)	1 (0.1%)	21 (0.7%)	<0.001
Primary	227 (36.4%)	358 (16.2%)	215 (28.0%)	800 (22.3%)	
Secondary	350 (61.5%)	1,538 (76.1%)	514 (71.4%)	2,402 (72.6%)	
More than secondary	0 (0.0%)	139 (7.2%)	3 (0.4%)	142 (4.5%)	
**Wealth quintiles**					
Lowest	232 (34.5%)	355 (14.3%)	213 (25.5%)	800 (20.2%)	<0.001
Second	125 (20.4%)	325 (14.6%)	177 (23.6%)	627 (17.6%)	
Middle	104 (19.3%)	352 (17.3%)	154 (22.6%)	610 (18.8%)	
Fourth	63 (13.2%)	422 (24.1%)	98 (14.5%)	583 (20.1%)	
Highest	64 (12.5%)	591 (29.7%)	92 (13.8%)	747 (23.3%)	
**Marital status**					
Not married	342 (60.6%)	1,186 (59.0%)	511 (71.7%)	2,039 (62.1%)	<0.001
Married	246 (39.4%)	859 (41.0%)	222 (28.3%)	1,327 (37.9%)	
**Age group**					
15-19	361 (64.8%)	815 (42.3%)	542 (77.1%)	1,718 (53.8%)	<0.001
20-24	227 (35.2%)	1,230 (57.7%)	192 (22.9%)	1,649 (46.2%)	

Mean (Standard deviation): p-value from linear regression.

Frequency (Percent%): p-value from Pearson test.

Statistics computed using the survey weights.

Tests adjusted for the survey design.

Supplemental Table 14. ZIMPHIA2020 (Zimbabwe) MALE Phone ownership, access and non-ownership characteristics

	No phone	Phone owner	Phone Access	Total	p-value
**N**	(N=519) (18.6%)	(N=1,520) (60.9%)	(N=543) (20.5%)	(N=2,582) (100.0%)	
**Age in years**	18.345 (2.845)	20.224 (2.439)	17.395 (2.355)	19.294 (2.773)	<0.001
**Gender**					
Male	519 (100.0%)	1,520 (100.0%)	543 (100.0%)	2,582 (100.0%)	
**Residence**					
Rural	447 (83.9%)	1,033 (65.0%)	468 (85.6%)	1,948 (72.7%)	<0.001
Urban	72 (16.1%)	487 (35.0%)	75 (14.4%)	634 (27.3%)	
**Highest school attended**					
No education	5 (1.1%)	6 (0.4%)	3 (0.5%)	14 (0.6%)	<0.001
Primary	223 (41.6%)	313 (19.1%)	171 (29.7%)	707 (25.5%)	
Secondary	287 (56.6%)	1,109 (74.0%)	367 (69.8%)	1,763 (69.9%)	
More than secondary	4 (0.7%)	91 (6.5%)	0 (0.0%)	95 (4.1%)	
**Wealth quintiles**					
Lowest	188 (33.9%)	245 (14.1%)	138 (23.4%)	571 (19.7%)	<0.001
Second	144 (27.5%)	314 (19.0%)	186 (34.0%)	644 (23.7%)	
Middle	98 (18.8%)	362 (23.9%)	122 (23.5%)	582 (22.9%)	
Fourth	48 (11.5%)	266 (20.1%)	46 (10.1%)	360 (16.4%)	
Highest	41 (8.3%)	333 (22.9%)	51 (9.0%)	425 (17.3%)	
**Marital status**					
Not married	476 (91.1%)	1,314 (86.3%)	525 (96.8%)	2,315 (89.4%)	<0.001
Married	41 (8.9%)	202 (13.7%)	17 (3.2%)	260 (10.6%)	
**Age group**					
15-19	370 (69.2%)	662 (40.1%)	457 (82.3%)	1,489 (54.2%)	<0.001
20-24	149 (30.8%)	858 (59.9%)	86 (17.7%)	1,093 (45.8%)	

Mean (Standard deviation): p-value from linear regression.

Frequency (Percent%): p-value from Pearson test.

Statistics computed using the survey weights.

Tests adjusted for the survey design.

Mean (Standard deviation): p-value from linear regression.
Frequency (Percent%): p-value from Pearson test.
Statistics computed using the survey weights.
Tests adjusted for the survey design.
